# A comparison of a single bout of stretching or foam rolling on range of motion in healthy adults

**DOI:** 10.1007/s00421-022-04927-1

**Published:** 2022-03-17

**Authors:** Andreas Konrad, Masatoshi Nakamura, Florian K. Paternoster, Markus Tilp, David G. Behm

**Affiliations:** 1 Institute of Human Movement Science, Sport and Health, Graz University, Graz, Austria; 2 Associate Professorship of Biomechanics in Sports, Technical University of Munich, Munich, Germany; 3 Institute for Human Movement and Medical Sciences, Niigata University of Health and Welfare, Niigata, Japan; 4 School of Human Kinetics and Recreation, Memorial University of Newfoundland, St. John’s, Newfoundland and Labrador, A1C 5S7 Canada

**Keywords:** Self-myofascial release, Foam roller, Flexibility, Extensibility, Healthy adults

## Abstract

**Purpose:**

Stretching and foam rolling are common warm-up exercises and can acutely increase the range of motion (ROM) of a joint. However, possible differences in the magnitude of change on ROM between these two interventions on the immediate and prolonged effects (e.g., 10 min after the intervention) are not yet well understood. Thus, the purpose of this review was to compare the immediate and prolonged effects of a single bout of foam rolling with a single bout of stretching on ROM in healthy participants.

**Methods:**

In total, 20 studies with overall 38 effect sizes were found to be eligible for a meta-analysis. For the main analysis, subgroup analysis, we applied a random-effect meta-analysis, mixed-effect model, respectively. The subgroup analyses included age groups, sex, and activity levels of the participants, as well as the tested muscles, the duration of the application, and the study design.

**Results:**

Meta-analyses revealed no significant differences between a single stretching and foam rolling exercise immediately after the interventions (ES = 0.079; *P* = 0.39) nor a difference 10 min (ES =  − 0.051; *P* = 0.65), 15 min (ES =  − 0.011; *P* = 0.93), and 20 min (ES =  − 0.161; *P* = 0.275) post-intervention. Moreover, subgroup analyses revealed no other significant differences between the acute effects of stretching and foam rolling (*P* > 0.05).

**Conclusion:**

If the goal is to increase the ROM acutely, both interventions can be considered as equally effective. Likely, similar mechanisms are responsible for the acute and prolonged ROM increases such as increased stretch tolerance or increased soft-tissue compliance.

## Introduction

Stretching with its varying techniques (i.e., static, ballistic, dynamic, and proprioceptive neuromuscular facilitation) (Magnusson et al. 1998) and foam rolling with or without vibration can acutely increase joint range of motion (ROM) (stretching: Behm and Chaouachi 2011; Behm et al. 2016, 2021b; Konrad et al. 2017b, 2019; Behm 2018; Konrad and Tilp 2020b, a) (foam rolling: Behm 2018; Behm and Wilke 2019; Wilke et al. 2020; Behm et al. 2020; Nakamura et al. 2021b, a; Yahata et al. 2021). Studies, which compared the acute effects of stretching and foam rolling on ROM, have either reported no difference between stretching and foam rolling (Halperin et al. 2014), a favorable effect of foam rolling on ROM compared to stretching (Su et al. 2017), or a favorable effect of stretching on ROM compared to foam rolling (Fairall et al. 2017). According to a meta-analysis (Wilke et al. 2020), the magnitude of the changes following stretching and foam rolling on ROM are similar [the difference between stretching and foam rolling: effect size (ES) = 0.02; 95% CI: − 0.67 to 0.73]. However, the main goal of Wilke et al. (2020) was to investigate the acute effects of foam rolling on ROM rather than comparing the acute effects of stretching and foam rolling on ROM. Hence, in the search code, the term “stretching” was not included and these authors might have overlooked some studies which investigated the acute effects of both stretching and foam rolling. Moreover, the search in the review of Wilke et al. (2020) was performed in February and March 2019 and only included nine comparative studies (foam rolling vs. stretching); hence, there is a need to update this meta-analysis with the recent and more expansive body of literature.

Apart from the immediate (i.e., acute) effects of foam rolling and stretching on ROM, the time course (i.e., prolonged effects) of the changes in ROM following these modalities is highly relevant for sports practice (i.e., time between stretching or foam rolling and the start of the competition or training). Whilst studies reported an increased ROM following stretching (Power et al. 2004; Konrad and Tilp 2020a) or foam rolling (Monteiro et al. 2018) for e.g., ≥30 min post-intervention, other studies showed no such changes up to that time point for both modalities (stretching: Kay et al. 2015; foam rolling: Nakamura et al. 2021b). Likely, the duration or intensity of the intervention may cause such contradicting findings. However, to get a better comparison on the time course effects on ROM between these two modalities (i.e., foam rolling vs. stretching), there is a need to perform a meta-analysis to clarify which intervention might cause a more prolonged effect for enhanced ROM.

Thus, the purpose of this review was to compare the immediate effects of a single bout of stretching versus a single bout of foam rolling on ROM in healthy participants. In addition, further goals were to compare the time course (i.e., 10, 15, and 20 min post-intervention) between single sessions of foam rolling and stretching on ROM and to summarize the mechanisms underlying ROM increases in both foam rolling and stretching based on the existing literature. According to the existing evidence, it was hypothesized that foam rolling compared to static stretching applied over the same duration would produce comparable immediate and time course effects in the increase in ROM.

Prolonged static stretching (>60 s per muscle group) in isolation (no aerobic or dynamic activities within the warm-up) has been reported to induce performance impairments (Behm and Chaouachi 2011; Kay and Blazevich 2012; Behm et al. 2016, 2021b). A recent meta-analysis (Konrad et al. 2021) reported a favorable effect on performance parameters for foam rolling when compared to static stretching but no such effect when foam rolling was compared to dynamic stretching. Additionally, when the rolling intervention was applied for more than 60 s, performance measures following foam rolling were more advantageous compared to stretching (Konrad et al. 2021). Hence, unlike static stretching, there was no duration threshold reported for foam rolling (Nakamura et al. 2021b). Thus, a validation of an alternative method for augmenting ROM without significant performance decrements such as foam rolling (Behm 2018; Behm and Wilke 2019; Wilke et al. 2020; Behm et al. 2020) could be beneficial to athletic or work performance.

## Materials and methods

This systematic review with meta-analysis was conducted according to the suggestions from Moher et al. (2009) and meets the PRISMA guidelines.

### Search strategy

**Fig. 1:**
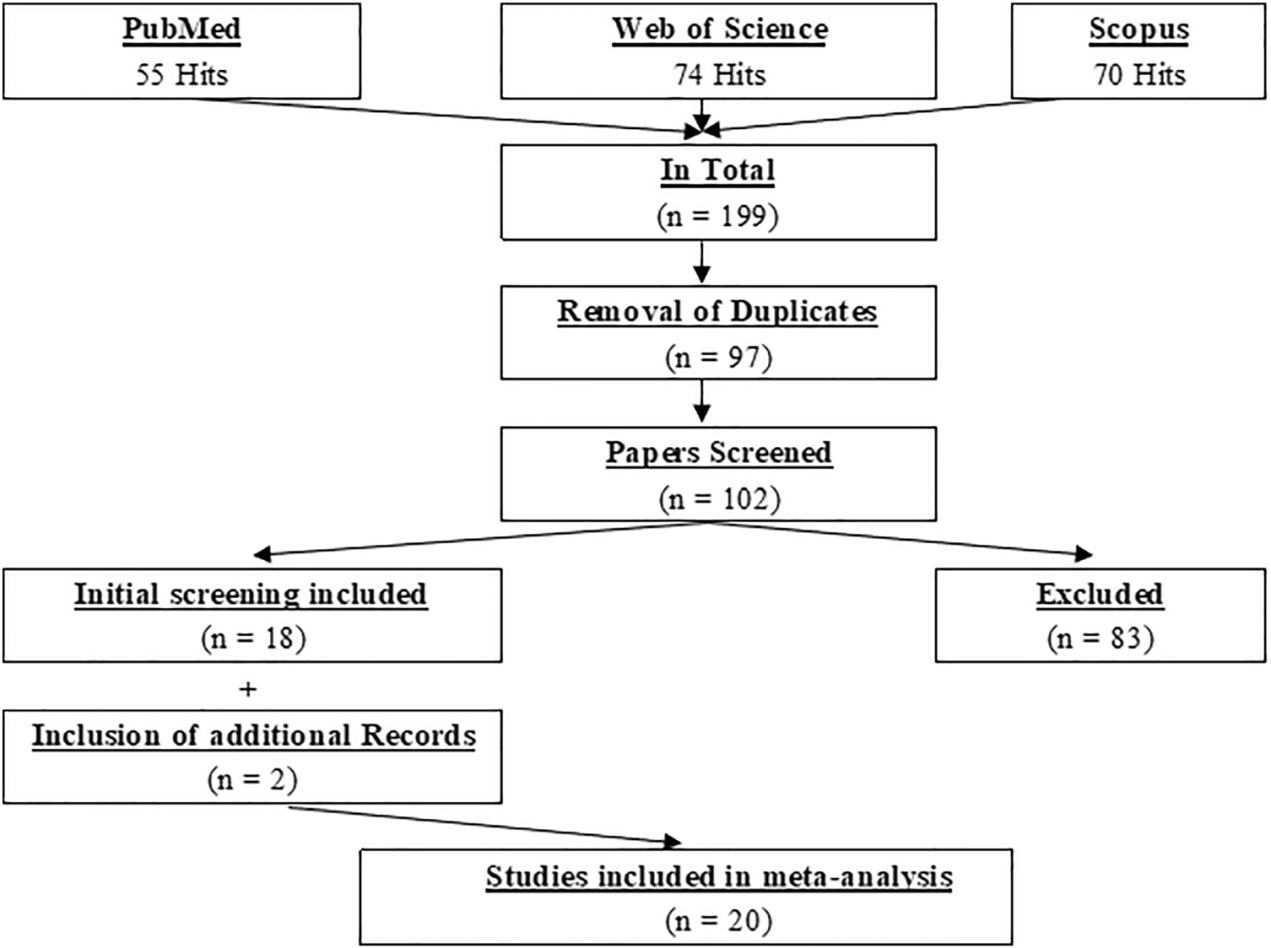
PRISMA flowchart

The electronic literature search was performed in three databases (i.e., PubMed, Scopus, and Web of Science) and the search period was until the 5th of November 2021. The keywords for the online search were the same for the various databases and were (“foam rolling” OR “self-myofascial release” OR “roller massage” OR “foam roller”) AND (stretch*). The systematic and independent search was conducted by three researchers (AK, FP, and MN). All the hits were screened by their title and abstract. If the eligibility of a paper remained unclear, the full text was further screened. Following this independent search, the findings of the researchers were compared. Disagreements were resolved by jointly reassessing the studies against the eligibility criteria. Following the removal of 97 duplicates in total, 102 papers were screened, where finally 18 papers found to be eligible for the meta-analysis. Additionally, two further papers from researchers’ libraries (AK, DB) were found to be eligible. No further eligible papers were found following an additional search of the references (search through the reference list) and citations (search through Google Scholar) of the already included papers. Consequently, in total, 20 papers (more than 2 × Wilke et al. (2020) meta-analysis) were included in the meta-analysis (see Fig. 1).

### Inclusion and exclusion criteria

This review included studies which compared the acute and/or the immediate effects of a single stretching exercise and single foam rolling exercise on ROM in healthy participants. We included studies written in all languages and either a crossover design (i.e., pre- to post-comparison or post-comparison) or parallel group design (pre- to post-comparison). However, we excluded studies with a parallel group design where only post-intervention values were compared. Moreover, we excluded studies which investigated combined effects of foam rolling and stretching. We further excluded conference papers or theses.

### Extraction of the data

From all the included papers, the characteristics of the participants (i.e., sex, activity level, and age), the sample size number, the study design (i.e., crossover, parallel design), the characteristics of the intervention (duration, stretching technique, vibration foam rolling vs. non-vibration foam rolling), the muscles tested by the ROM test, and the pre- and post-intervention values plus standard deviation of the main variable ROM of both groups (foam rolling, stretching) were extracted. If the full paper did not provide all the data required for the meta-analysis, the corresponding authors were contacted via email and Research Gate.

### Statistics and data synthesis

**Fig. 2:**
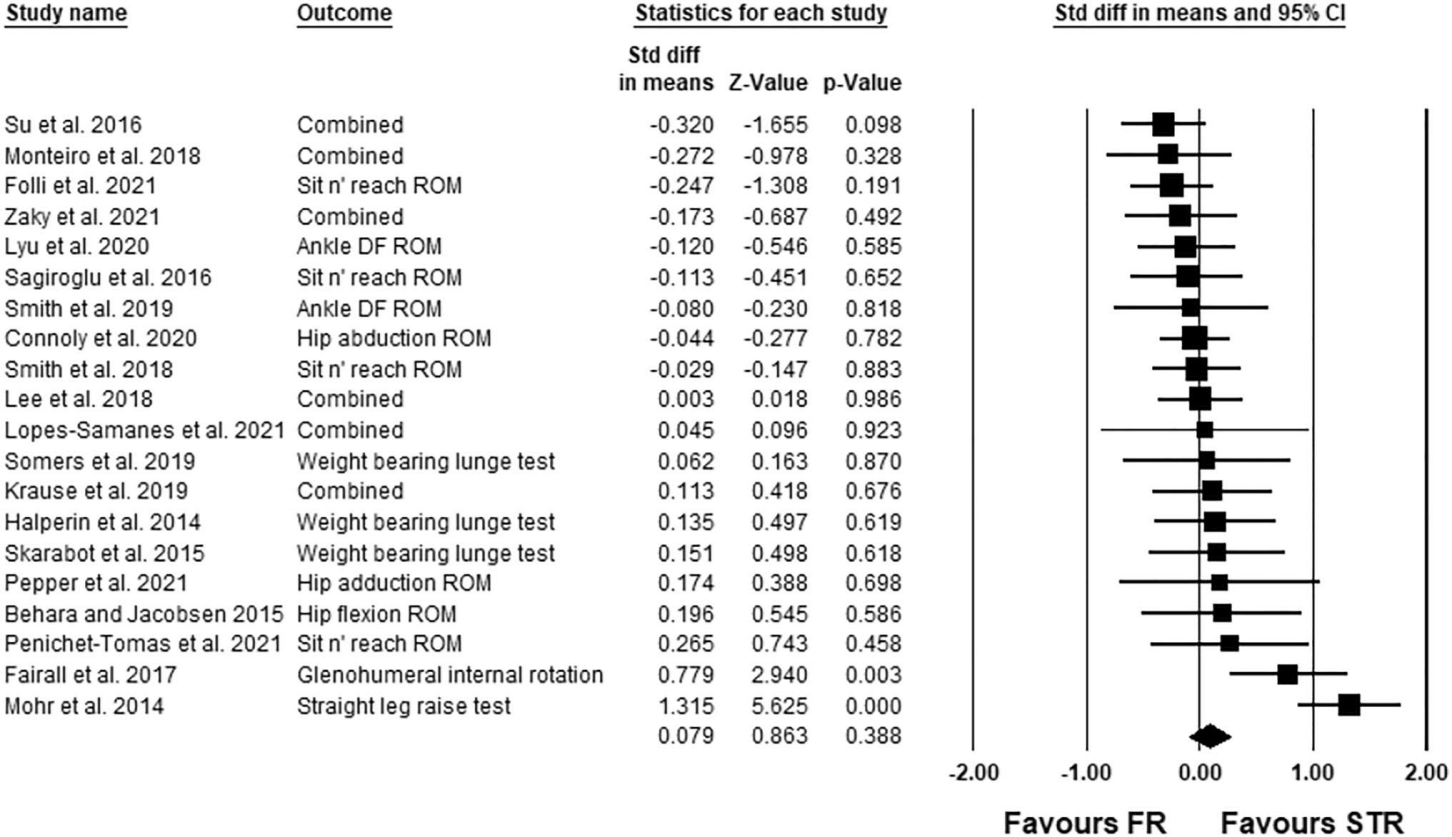
Forest plot presenting the 20 included studies with overall 38 effect sizes. Std diff in means = standardized difference in means; CI = confidence interval; FR = foam rolling; STR = stretching; combined = mean of the selected outcomes of one study

The meta-analysis was conducted using the Comprehensive Meta-Analysis (CMA) software according to the suggestions of Borenstein et al. (2009). Consequently, a random-effect meta-analysis was used to assess the effect size (standardized mean difference) for the immediate and the time course effects. If any study reported more than one effect size, as suggested by Borenstein et al., (2009) the mean of all the outcomes (effect sizes) within one study was used for the analysis and defined with the term “combined” (see, i.e., Fig. 2). Although there is no general rule of thumb (Borenstein et al. 2009), we only performed a meta-analysis when ≥3 studies could be included in the respective analysis. Hence, the time course effects of 10, 15, and 20 min post-stretching could be assessed. Moreover, using a mixed-effect model, we conducted various subgroup analyses with age of the participants (i.e., ≤23.4 vs >23.4 years; 23.4 = average age of all participants in this meta-analysis), activity level of the participants (sedentary/physical active vs. well trained/professional), tested muscle by the ROM test (hamstrings, quadriceps, triceps surae, shoulder), duration of the application (i.e., ≤60 s; >60 s,), sex (i.e., mixed/female vs male), stretching technique (static stretching, dynamic stretching), and the study design (parallel design, crossover). Since only one out of the 20 studies included female subjects, we formed the subgroups mixed/female and male. Subgroup analysis on the time course effects was not possible, because only less than three studies (i.e., per subgroup) were available. Q-statistics were applied (Borenstein et al. 2009) to determine if there were differences between the effect sizes of the subgroups. Hopkins et al. (2009) suggested to define the standardized mean difference of <0.2, 0.2–0.6, 0.6–1.2, 1.2–2.0, 2.0–4.0, and >4.0 as trivial, small, moderate, large, very large, and extremely large, respectively. To assess the heterogeneity *I*^2^ statistics were calculated among the effect sizes, and thresholds of 25%, 50%, and 75% were defined as having a low, moderate, and high level of heterogeneity, respectively (Higgins et al. 2003; Behm et al. 2021a). An alpha level of 0.05 was defined for the statistical significance of all the tests.

### Risk-of-bias assessment and methodological quality

To assess the methodological quality of the included studies, the PEDro scale was used. Two independent researchers (AK, MN) assessed 11 methodological issues by assigning with either one or no point. Note that studies with a higher score represent a higher methodological quality. If any conflict between the ratings of the two researchers was found, the methodological issues were reassessed and discussed. Moreover, to assess a possible publication bias, the statistics of the Egger’s regression intercept test was used.

## Results

### Results of the search

In total, 20 studies investigated the immediate effects of both a single foam rolling exercise and a single stretching exercise on ROM, and overall, 38 effect sizes were included for this meta-analysis. Additionally, out of these 20 studies, the time course effects of 10, 15, and 20 min post-intervention were investigated by five, four, and three studies, respectively. In summary, 411 participants with a mean age of 23.4 (±4.9 years) participated in the included studies. Moreover, Table 1 presents the characteristics and outcome variables of these studies.

**Table 1:** Participants’ characteristics, details of the interventions, and the outcomes of the included studies (*n* = 20)

Study	Participants	Type of stretching	Type of foam rolling	Intervention duration per muscle group	Outcome
Smith et al. (2018)	*N* = 29 sedentary to physically active males and females (age = 22 ± 3)	Dynamic	Non-vibration	90 s for foam rolling/dynamic stretching, duration per muscle group not clear	Sit and Reach
Behara and Jacobson (2017)	*N* = 12 well trained male Division 1 offensive lineman (age 20.0 ± 1.41)	Dynamic	Non-vibration	60 s for foam rolling/dynamic stretching, duration per muscle group not clear	Hip flexion ROM
Su et al. (2017)	*N* = 30 physically active males and females (21.43 ± 1.5)	Static dynamic	Non-vibration	90 s foam rolling and static stretching; 180 s dynamic stretching	Sit-and-reach modified Thomas Test
Fairall et al. (2017)	*N* = 12 male amateur softball players (age 36.92 ± 11.17)	Static	Non-vibration	120 s stretching; 180 s foam rolling	Glenohumeral internal rotation ROM
Škarabot et al. (2015)	*N* = 11 highly trained male and female swimmers (age 15.3 ± 1)	Static	Non-vibration	90 s	Weight-bearing lunge test
Lee et al. (2018)	*N* = 30 male college students (age 20.4 ± 1.2)	Static	Vibration non-vibration	90 s	Leg extension ROM leg flexion ROM
Lyu et al. (2020)	*N* = 20 male recreational active (age 21 ± 1.01)	Static	Vibration	90 s	Ankle dorsiflexion ROM
Folli et al. (2021)	*N* = 29 male and female healthy athletes (age 16 ± 1.23)	Static	Non-vibration	60 s	Sit-and-reach
Penichet-Tomas et al. (2021)	*N* = 8 male well trained rowers (24.8 ± 3.8)	Static	Non-vibration	90 s	Sit-and-reach
Lopez-Samanes et al. (2021)	*N* = 11 elite male tennis players (age 20.64 ± 3.56)	Dynamic	Non-vibration	60 s for foam rolling/dynamic stretching seconds per muscle group not clear	Straight leg raise test
Connolly et al. (2020)	*N* = 40 males and females (activity level not stated) (males age 22.5 ± 1.8; Females age 23.6 ± 4.2)	Static	Non-vibration	60 s	Hip abduction ROM
Zaky et al. (2021)	*N* = 20 elite female handball players (age 22.83 ± 1.52)	Dynamic	Non-vibration	60 s for foam rolling/dynamic stretching seconds per muscle group not clear	Shoulder flexion ROM shoulder extension ROM Shoulder internal rotation ROM Shoulder external rotation ROM
Krause et al. (2018)	*N* = 16 males and females (activity level not stated) (males age 31.2 ± 4.8; Females age 33.5 ± 5.6)	Static	Non-vibration	120 s	Active knee flexion passive knee flexion
Sagiroglu et al. (2017)	*N* = 16 male well trained combat athletes (age 23.9 ± 3.70)	Static	Non-vibration	60 s	Sit-and-reach
Halperin et al. (2014)	*N* = 14 male and female recreational active (males age 23 ± 4; Females age 22 ± 3)	Static	Non-vibration	90 s	Weight-bearing lunge test
Monteiro et al. (2018)	*N* = 12 male and female recreational active (age 27.88 ± 3.23)	Static PNF	non-vibration	60 s or 120 s	Shoulder flexion ROM Shoulder extension ROM
Somers et al. (2020)	N = 28 male and female physically active (Age foam rolling group 26.07 ± 4.83; Age stretching group 26.86 ± 4.75)	Dynamic	Non-vibration	120 s	Weight-bearing lunge test
Smith et al. (2019)	*N* = 33 males and females (activity level not stated) (males age 21.7 ± 1.7; females age 21.3 ± 2.0)	Static	Non-vibration	90 s	Ankle dorsiflexion ROM
Pepper et al. (2021)	N = 20 males and females (activity level not stated) (Age foam rolling group 27.1 ± 6.5; Age stretching group 26.7 ± 8.6)	PNF	Non-vibration	60 s	Hip adduction ROM
Mohr et al. (2014)	*N* = 20 recreational active (gender not stated) (Age foam rolling group 21.00 ± 2.21; Age stretching group 21.2 ± 2.44)	Static	Non-vibration	180 s	Straight leg raise test

*PNF* proprioceptive neuromuscular facilitation, *ROM* range of motion

### Risk-of-bias assessment and methodological quality

Egger’s regression intercept test for the immediate effects (intercept 1.16; *P* = 0.38) but also for the time course effects 10 min post-intervention (intercept − 0.04; *P* = 0.97), 15 min post-intervention (intercept 0.72; *P* = 0.14), and 20 min post-intervention (intercept − 1.82; *P* = 0.65) indicate that no reporting bias is likely.

Moreover, a low risk of bias was indicated with an average PEDro score of 6.6 (±1.1; range 4–9). Both assessors agreed with 95.9% of the overall 220 (20 studies × 11 criteria) criteria. However, the mismatches were discussed and the assessors finally agreed with the scores presented in Table 2.

**Table 2:** PEDro scale of the included studies; * = was not counted for the final score; 1 = one point awarded; 0 = no point awarded

Study	Inclusion criteria	Random allocation	Concealed allocation	Similarity at baseline	Subject blinding	Therapist blinding	Assessor blinding	>85% follow-up	Intention to treat analysis	Between-group comparison	Point estimates and variability	Total
Smith et al. (2018)	1	1	0	1	1	0	0	1	1	1	1	7
Behara and Jacobson (2017)	1	1	0	1	1	0	0	1	1	1	1	7
Su et al. (2017)	1	1	0	1	1	0	0	1	1	1	1	7
Fairall et al. (2017)	1	1	0	1	0	0	0	1	1	1	1	6
Škarabot et al. (2015)	1	1	1	1	0	0	0	1	1	1	1	7
Lee et al. (2018)	1	1	0	1	1	0	0	1	1	1	1	7
Lyu et al. (2020)	1	1	0	1	1	0	0	1	1	1	1	7
Folli et al. (2021)	1	1	0	1	1	0	0	1	1	1	1	7
Penichet-Tomas et al. (2021)	1	0	0	1	0	0	0	1	1	1	0	4
Lopez-Samanes et al. (2021)	1	1	0	1	1	0	0	1	1	1	1	7
Connolly et al. (2020)	1	1	0	1	1	0	0	1	1	1	1	7
Zaky et al. (2021)	0	0	0	1	0	0	0	1	1	1	0	4
Krause et al. (2018)	1	1	0	1	0	0	0	1	1	1	1	6
Sagiroglu et al. (2017)	1	1	0	1	1	0	0	1	1	1	1	7
Halperin et al. (2014)	1	1	0	1	1	0	0	1	1	1	1	7
Monteiro et al. (2018)	1	1	0	1	0	0	1	1	1	1	0	6
Somers et al. (2020)	1	1	1	1	0	1	1	1	1	1	1	9
Smith et al. (2019)	0	1	1	1	0	1	1	1	0	1	0	7
Pepper et al. (2021)	1	1	0	1	0	1	1	1	1	1	0	7
Mohr et al. (2014)	1	1	0	1	0	0	0	1	1	1	1	6

### Main analysis for the immediate effects

The average percentage increase in ROM in the included studies following stretching, foam rolling was 7.2 ± 8.7%, 7.2 ± 5.5%, respectively. The meta-analysis revealed no significant difference between the two modalities (ES = 0.079; Z = 0.863; CI (95%) − 0.101 to 0.259; *P* = 0.39; *I*^2^ = 60.18). Moreover, Fig. 2 presents the forest plot of the meta-analysis, sorted from the lowest to the highest effect size.

### Subgroup analysis for the immediate effects

A summary of all the subgroup analyses is shown in Table 3. None of the analysis showed any significant difference between the subgroups as the age of the participants (i.e., ≤23.4 vs >23.4 years) (*Q* = 0.35; *P* = 0.56), activity level of the participants (sedentary/physical active vs. well trained/professional) (*Q* = 0.39; *P* = 0.53), tested muscle by the ROM test (hamstrings, quadriceps, triceps surae, shoulder) (*Q* = 0.57; *P* = 0.90), duration of the application (i.e., >60 s, ≤60 s) (*Q* = 1.99; *P* = 0.16), sex (i.e., mixed/female vs. male) (*Q* = 2.23; *P* = 0.14), stretching technique (static stretching, dynamic stretching) (*Q* = 2.05; *P* = 0.15), and the study design (parallel design, crossover) (*Q* = 1.16; *P* = 0.28).

**Table 3:** Statistics of the subgroup analysis

Subgroup	Number of measures	Std diff in means (95% CI)	*P* value	*Q-*statistics
Age of the participants				
≤23.4 years	13	0.05 (− 0.176 to 0.283)	0.65	
>23.4 years	7	0.14 (− 0.142 to 0.424)	0.33	
Overall	20	0.09 (− 0.090 to 0.267)	0.33	(*Q* = 0.22; df (*Q*) = 1; *P* = 0.64)
Activity level of the participants				
Sedentary/physical active	15	0.1 (− 0.125 to 0.325)	0.38	
Well trained/professional	5	− 0.01 (− 0.276 to 0.254)	0.94	
Overall	20	0.05 (− 0.118 to 0.225)	0.54	(*Q* = 0.39; df (Q) = 1; P = 0.53)
Muscle tested				
Hamstrings	9	0.11 (− 0.224 to 0.451)	0.51	
Quadriceps	4	− 0.54 (− 0.376 to 0.268)	0.74	
Triceps surae	5	0.013 (− 0.240 to 0.265)	0.92	
Shoulder	3	0.112 (− 0.542 to 0.766)	0.74	
Overall	21	0.026 (− 0.150 to 0.009)	0.76	(*Q* = 0.57; df (*Q*) = 3; *P* = 0.90)
Duration of the intervention				
≤60 s	5	− 0.14 (− 0.328 to 0.059)	0.17	
>60 s	11	0.12 (− 0.175 to 0.409)	0.43	
Overall	16	− 0.06 (− 0.219 to 0.103)	0.48	(*Q* = 1.99; df (*Q*) = 1; *P* = 0.16)
Sex				
Male	7	0.13 (− 0.122 to 0.376)	0.32	
Mixed/female	12	− 0.09 (− 0.225 to 0.047)	0.19	
Overall	19	− 0.04 (− 0.159 to 0.080)	0.51	(*Q* = 2.23; df (*Q*) = 1; *P* = 0.14)
Stretching technique				
Static stretching	6	− 0.13 (− 0.341 to 0.077)	0.22	
Dynamic stretching	14	0.1 (− 0.138 to 0.336)	0.41	
Overall	19	− 0.03 (− 0.188 to 0.126)	0.7	(*Q* = 2.05; df (*Q*) = 1; *P* = 0.15)
Study design				
Crossover	16	− 0.03 (− 0.148 to 0.097)	0.68	
Parallel	4	0.4 (− 0.370 to 1.178)	0.31	
Overall	20	0.015 (− 0.136 to 0.106)	0.81	(*Q* = 1.16; df (*Q*) = 1; *P* = 0.28)

Negative values of Std diff (= standardized difference) in means indicate a favorable effect for foam rolling (and vice versa).

### Main analysis for the time course effects

The average percentage increase in ROM 10 min post-intervention in the included studies following stretching, foam rolling was 6.7 ± 3.6%, 7.6 ± 4.8%, respectively. Moreover, the average percentage increase in ROM 15 min post-intervention following stretching, foam rolling was 11.6 ± 7.0%, 10.5 ± 5.6%, respectively. Finally, the ROM 20 min post-intervention was 4.5 ± 3.7%, 5.9 ± 3.6%, for stretching, foam rolling, respectively. The meta-analyses on the time course revealed no significant difference between the modalities at 10 min, 15 min, 20 min post-intervention with an effect size of − 0.051 (Z =  − 0.448; CI (95%) − 0.274 to 0.172; *P* = 0.65; *I*^2^ = 0.00), − 0.011 (*Z* =  − 0.083; CI (95%) − 0.266 to 0.244; *P* = 0.93; *I*^2^ = 0.00), − 0.161 (*Z *=  − 1.092; CI (95%) − 0.451 to 0.128; *P* = 0.275; *I*^2^ = 0.00), respectively.

**Fig. 3:**
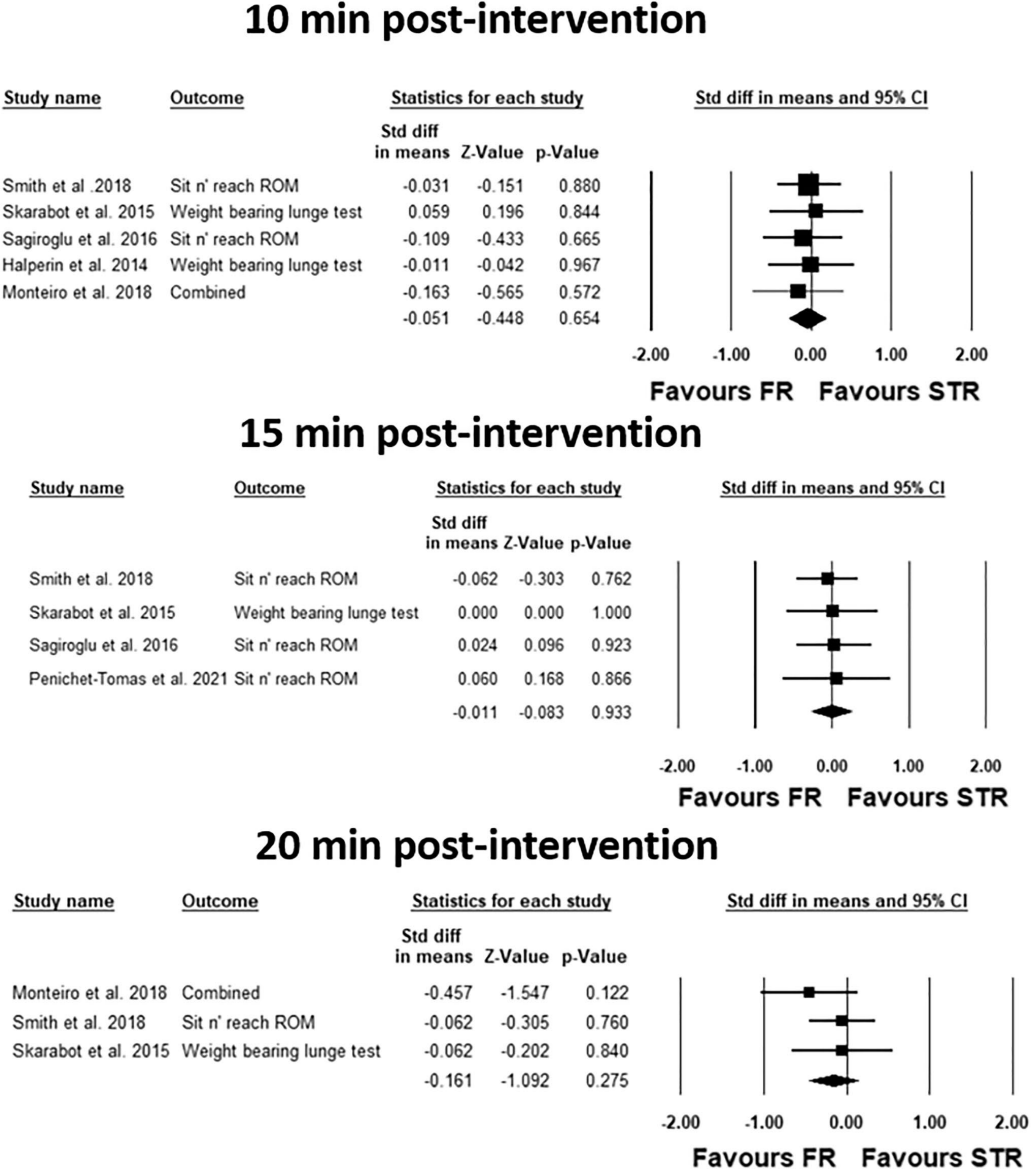
Forest plots presenting the time course effects 10 min, 15 min, and 20 min post-intervention. Std diff in means = standardized difference in means; CI = confidence interval; FR = foam rolling; STR = stretching; combined = mean of the selected outcomes of one study

Moreover, Fig. 3 presents the forest plots of the meta-analyses (i.e., 10 min, 15 min, and 20 min).

## Discussion

The purpose of this review was to compare the immediate and time course effects of a single foam rolling and stretching exercise on ROM. The results revealed no significant difference between the two modalities immediately after the interventions (ES = 0.079; *P* = 0.39) nor 10 min (ES = − 0.051; *P* = 0.65), 15 min (ES = − 0.011; *P* = 0.93), and 20 min (ES = − 0.161; *P* = 0.28) post-intervention. Moreover, subgroup analyses revealed no differences (*P* > 0.05) between the age groups of the participants (i.e., ≤23.4 vs >23.4 years), activity levels of the participants (sedentary/physical active vs. well trained/professional), tested muscle by the ROM test (hamstrings, quadriceps, triceps surae, shoulder), duration of the application (i.e., >60 s, ≤60 s), sex (i.e., mixed/female vs. male), stretching technique (static stretching, dynamic stretching), and the study design (parallel design, crossover).

Similarly, to Wilke et al. (2020), we have not found a difference in the immediate (i.e., acute) effect on ROM between stretching and foam rolling. However, in the meta-analysis of Wilke et al. (2020), the main focus was on investigating the acute effects of foam rolling on ROM rather than comparing the acute effects of stretching and foam rolling on ROM. Hence, in the search code of Wilke et al. (2020), the term “stretching” was not included and the search was performed up to February 2019. Consequently, Wilke et al. (2020) found nine studies to be eligible to compare the acute effects of foam rolling and stretching, whilst we found a further 11, and hence, in total 20 studies to be eligible. Although the results were similar, we believe that including approximately twofold more studies in our meta-analysis strengthens the evidence that stretching and foam rolling can be considered equally to increase the ROM. Additionally, our meta-analysis was the first which compared the time course effects (i.e., 10 min, 20 min, 30 min post-intervention) of foam rolling and stretching on ROM. Similar to the immediate effects, no differences were found between the modalities.

**Fig. 4:**
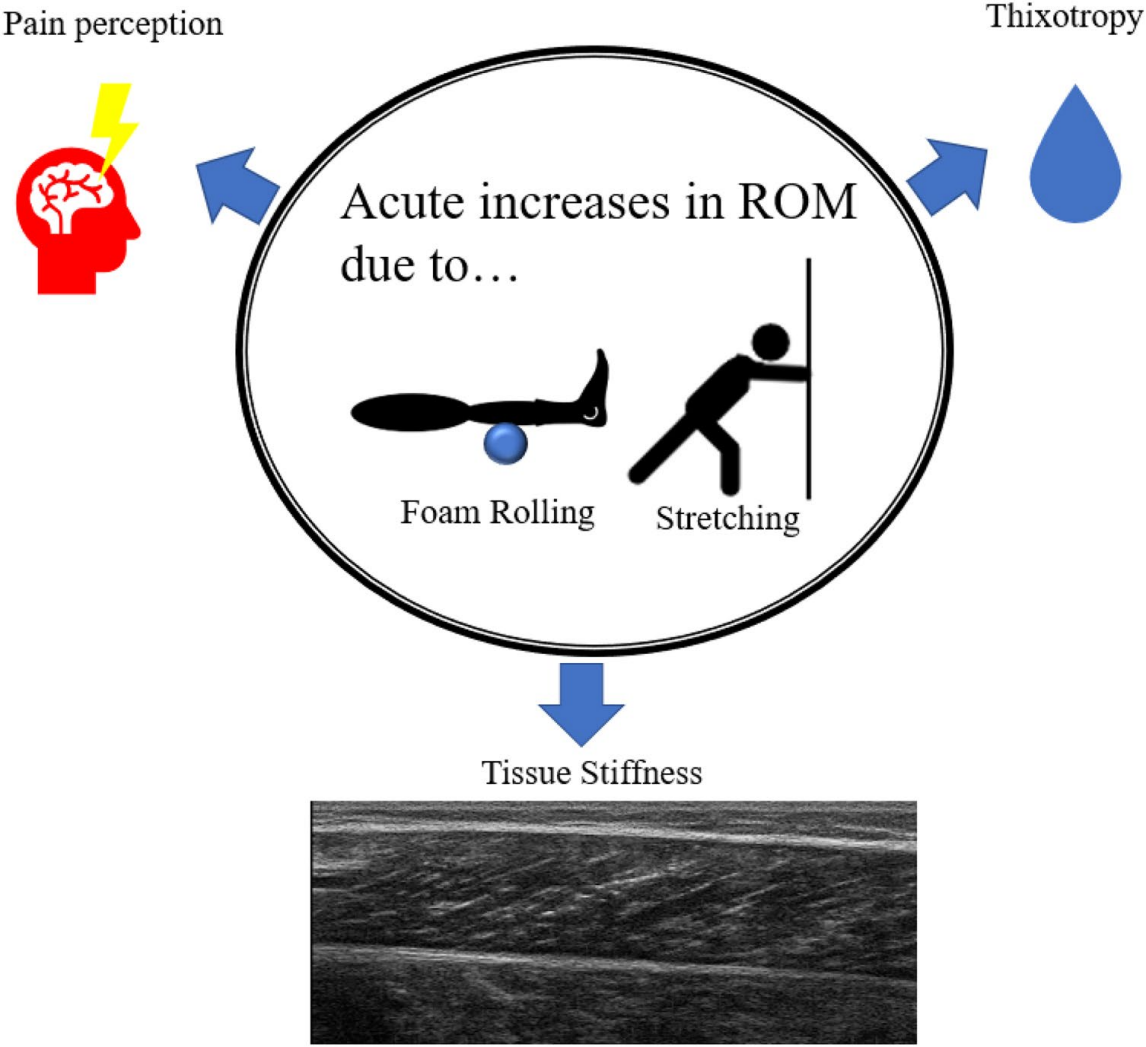
Potential mechanism for acute increase in range of motion (ROM) following a single bout of stretching or foam rolling

Various studies have reported that stretching (Behm and Chaouachi 2011; Behm et al. 2016, 2021b; Konrad et al. 2017b, 2019; Behm 2018; Konrad and Tilp 2020b,a) and foam rolling (Behm 2018; Behm and Wilke 2019; Wilke et al. 2020; Behm et al. 2020; Nakamura et al. 2021b, a; Yahata et al. 2021) can increase the ROM of a joint acutely. However, the mechanism behind such an increase in ROM in both stretching and foam rolling is debated. Following a single bout of stretching, the acute increase in ROM is often associated with a decrease in soft-tissue stiffness (muscle: Kay et al. 2015; Konrad et al. 2019); tendon: (Kubo et al. 2001; Kato et al. 2010)) and/or changes in the tolerance to stretch (i.e., pain perception) (Magnusson et al. 1996; Konrad et al. 2017a). Similarly, in foam rolling, the acute increases in ROM may be attributed to decreased muscle stiffness (Behm 2018; Behm and Wilke 2019; Reiner et al. 2021b) or an increased stretch tolerance (Nakamura et al. 2021b). Additionally, thixotropic effects might be related to the increase in ROM following foam rolling (Behm and Wilke 2019) as well as with stretching (Behm 2018). The applied friction or tension on the treated muscle, skin, and fascia could have an impact on fluid viscosity and, hence, lead to less resistance to a movement (Behm 2018; Behm and Wilke 2019). Bringing these findings together, it is likely that similar mechanisms are responsible for the acute (immediate) increase in ROM following both foam rolling and stretching (see Fig. 4). Hence, this might also explain that there was no difference in the magnitude of change between the two interventions in our meta-analysis. Concerning the time course effects, it was reported that a decrease in muscle stiffness following an acute bout of stretching was returned to baseline after five minutes (Mizuno et al. 2013; Konrad et al. 2019; Konrad and Tilp 2020b), although the ROM was reported to be increased up to 120 min with a similar protocol (Power et al. 2004). Hence, there seems to be evidence that functional changes (e.g., increase in ROM) last longer compared to structural changes (e.g., decrease in tissue stiffness). Consequently, other mechanism than tissue stiffness such as thixotropic effects (Behm 2018) or changes in tolerance to stretch or pain (Magnusson et al. 1996) likely play a role for the continuing increase in ROM especially following stretching exercises and, hence, probably following foam rolling, as well.

There are always limitations to any investigation. Future studies need to explore the effects of a greater range of rolling durations as the present selection of studies primarily employed 60 s (8 studies) to 90 s (8 studies) of rolling with only two studies each intervening with 120 s or 180-s of rolling. It would be of interest to note whether shorter or longer durations provide similar increases in ROM. Furthermore, 5/20 studies tested ROM with the sit-and-reach test. The ROM measured in the sit-and-reach test can be attenuated by a restrictive lower back even if the rolling had favourable effects on the hamstrings extensibility. Hence, the rolling-induced effects on hamstrings extensibility with this type of hip flexion testing could have been underestimated. Only 3/20 studies monitored upper body ROM (i.e., shoulders) and thus the extent of possible rolling-induced ROM differences may be influenced by anatomical location and should be further considered. As is typical of exercise or sport science research, the predominant mean age of the participants was between 20 and 27 years with only two studies each examining participants with an average age between 31–37 and 15–16 years respectively. Additional studies are suggested to examine youth at different stages of peak height velocity (pubescence) as well middle-aged and elderly populations. Furthermore, the vast majority of foam rolling-related studies report on the effects of rolling on ROM and performance but very few evaluate mechanisms (e.g., Krause et al. 2018; Nakamura et al. 2021a; Reiner et al. 2021b; Pepper et al. 2021). As three of the four studies that reported on mechanisms were published within the last year, it is hoped that this is a sign of a trend for more research involving mechanistic measures.

Based on an integration of Konrad et al. (2021) (i.e., favorable effects on performance for foam rolling when compared to static stretching) and the present findings, we would rather recommend foam rolling than prolonged static stretching in isolation (i.e., no additional dynamic activities) as a warm-up when flexibility and performance should be optimized. However, it has to be noted that, when post-stretching dynamic activities are performed following static, dynamic stretching, or proprioceptive neuromuscular facilitation (PNF) for stretching durations up to 120 s, no negative or positive effect on performance was reported (Samson et al. 2012; Blazevich et al. 2018; Reid et al. 2018; Reiner et al. 2021a).

## Conclusion

The present review revealed no difference between a single bout of stretching and foam rolling exercise immediately after the interventions but also 10, 15, and 20 min post-intervention. Neither of the subgroup analyses revealed a significant difference between the acute effects of stretching and foam rolling such as the age groups of the participants (i.e., ≤25 vs >25 years), activity levels of the participants (sedentary/physical active vs. well trained/professional), tested muscle by the ROM test (hamstrings, quadriceps, triceps surae, shoulder), duration of the application (i.e., >60 s, ≤60 s), sex (i.e., mixed/female vs. male), stretching technique (static stretching, dynamic stretching), and the study design (parallel design, crossover). Hence, if the goal is to increase the ROM acutely, both interventions can be considered equally effective. However, foam rolling rather than isolated static stretching without aerobic or dynamic activities within the warm-up should be implemented as a warm-up when ROM and performance (e.g., jumping) are equally important determinants for the subsequent training or competition. Likely, similar mechanisms are responsible for the acute and prolonged ROM increases such as increased stretch tolerance or soft-tissue compliance.

## Abbreviations

ES: Effect size
PNF: Proprioceptive neuromuscular facilitation
ROM: Range of motion

## References

[CR1] Behara B, Jacobson BH (2017). Acute effects of deep tissue foam rolling and dynamic stretching on muscular strength, power, and flexibility in Division I Linemen. J Strength Cond Res.

[CR2] Behm DG (2018). The science and physiology of flexibility and stretching.

[CR3] Behm DG, Alizadeh S, Anvar SH (2021). Non-local acute passive stretching effects on range of motion in healthy adults: a systematic review with meta-analysis. Sport Med.

[CR4] Behm DG, Alizadeh S, Hadjizadeh Anvar S (2020). Foam rolling prescription: a clinical commentary. J Strength Cond Res.

[CR5] Behm DG, Blazevich AJ, Kay AD, McHugh M (2016). Acute effects of muscle stretching on physical performance, range of motion, and injury incidence in healthy active individuals: a systematic review. Appl Physiol Nutr Metab.

[CR6] Behm DG, Chaouachi A (2011). A review of the acute effects of static and dynamic stretching on performance. Eur J Appl Physiol.

[CR7] Behm DG, Kay AD, Trajano GS, Blazevich AJ (2021). Mechanisms underlying performance impairments following prolonged static stretching without a comprehensive warm-up. Eur J Appl Physiol.

[CR8] Behm DG, Wilke J (2019). Do self-myofascial release devices release myofascia? rolling mechanisms: a narrative review. Sport Med.

[CR9] Blazevich A, Gill ND, Kvorning T (2018). No effect of muscle stretching within a full, dynamic warm-up on athletic performance. Med Sci Sport Exerc.

[CR10] Borenstein M, Hedges LV, Higgins JPT, Rothstein HR (2009). Introduction to meta-analysis.

[CR11] Connolly G, Hammer RL, Powell JA, O’connor PL (2020). A single bout of foam rolling increases flexibility of the hip adductor muscles without compromising strength. Int J Exerc Sci.

[CR12] Fairall RR, Cabell L, Boergers RJ, Battaglia F (2017). Acute effects of self-myofascial release and stretching in overhead athletes with GIRD. J Bodyw Mov Ther.

[CR13] Folli A, Ghirlanda F, Cescon C (2021). A single session with a roller massager improves hamstring flexibility in healthy athletes: a randomized placebo-controlled crossover study. Sport Sci Health.

[CR14] Halperin I, Aboodarda SJ, Button DC, Andersen LL, Behm DG (2014). Roller massager improves range of motion of plantar flexor muscles without subsequent decreases in force parameters. Int J Sport Phys Ther.

[CR15] Higgins JPT, Thompson SG, Deeks JJ, Altman DG (2003). Measuring inconsistency in meta-analyses. Br Med J.

[CR16] Hopkins WG, Marshall SW, Batterham AM, Hanin J (2009). Progressive statistics for studies in sports medicine and exercise science. Med Sci Sport Exerc.

[CR17] Kato E, Kanehisa H, Fukunaga T, Kawakami Y (2010). Changes in ankle joint stiffness due to stretching: the role of tendon elongation of the gastrocnemius muscle. Eur J Sport Sci.

[CR18] Kay AD, Blazevich AJ (2012). Effect of acute static stretch on maximal muscle performance: a systematic review. Med Sci Sport Exerc.

[CR19] Kay AD, Husbands-Beasley J, Blazevich AJ (2015). Effects of contract-relax, static stretching, and isometric contractions on muscle-tendon mechanics. Med Sci Sports Exerc.

[CR20] Konrad A, Budini F, Tilp M (2017). Acute effects of constant torque and constant angle stretching on the muscle and tendon tissue properties. Eur J Appl Physiol.

[CR21] Konrad A, Reiner MM, Thaller S, Tilp M (2019). The time course of muscle-tendon properties and function responses of a five-minute static stretching exercise. Eur J Sport Sci.

[CR22] Konrad A, Stafilidis S, Tilp M (2017). Effects of acute static, ballistic, and PNF stretching exercise on the muscle and tendon tissue properties. Scand J Med Sci Sport.

[CR23] Konrad A, Tilp M (2020). The acute time course of muscle and tendon tissue changes following one minute of static stretching. Curr Issues Sport Sci.

[CR24] Konrad A, Tilp M (2020). The time course of muscle-tendon unit function and structure following three minutes of static stretching. J Sport Sci Med.

[CR25] Konrad A, Tilp M, Nakamura M (2021). A comparison of the effects of foam rolling and stretching on physical performance. A systematic review and meta-analysis. Front Physiol.

[CR26] Krause F, Wilke J, Niederer D (2018). Acute effects of foam rolling on passive stiffness, stretch sensation, and fascial sliding: a randomized controlled trial. J Bodyw Mov Ther.

[CR27] Kubo K, Kanehisa H, Kawakami Y, Fukunaga T (2001). Influence of static stretching on viscoelastic properties of human tendon structures in vivo. J Appl Physiol.

[CR28] Lee CL, Chu IH, Lyu BJ (2018). Comparison of vibration rolling, nonvibration rolling, and static stretching as a warm-up exercise on flexibility, joint proprioception, muscle strength, and balance in young adults. J Sports Sci.

[CR29] Lopez-Samanes A, Del Coso J, Hernández-Davó JL (2021). Acute effects of dynamic versus foam rolling warm-up strategies on physical performance in elite tennis players. Biol Sport.

[CR30] Lyu BJ, Lee CL, Chang WD, Chang NJ (2020). Effects of vibration rolling with and without dynamic muscle contraction on ankle range of motion, proprioception, muscle strength and agility in young adults: a crossover study. Int J Environ Res Public Health.

[CR31] Magnusson SP, Aagard P, Simonsen E, Bojsen-Møller F (1998). A biomechanical evaluation of cyclic and static stretch in human skeletal muscle. Int J Sports Med.

[CR32] Magnusson SP, Simonsen EB, Aagaard P (1996). Mechanical and physiological responses to stretching with and without preisometric contraction in human skeletal muscle. Arch Phys Med Rehabil.

[CR33] Mizuno T, Matsumoto M, Umemura Y (2013). Viscoelasticity of the muscle-tendon unit is returned more rapidly than range of motion after stretching. Scand J Med Sci Sports.

[CR34] Moher D, Liberati A, Tetzlaff J, Altman DG (2009). Preferred reporting items for systematic reviews and meta-analyses: the PRISMA statement. PLoS Med.

[CR35] Mohr AR, Long BC, Goad CL (2014). Effect of foam rolling and static stretching on passive hip-flexion range of motion. J Sport Rehabil.

[CR36] Monteiro ER, Wakefield B, Ribeiro MS (2018). Anterior and posterior thigh self-massage and stretching acutely increases shoulder range-of-motion/Automassagem e alongamento nas regioes anterior e posterior de coxa aumentam de forma aguda a amplitude articular de ombro. Motricidade.

[CR37] Nakamura M, Konrad A, Kiyono R (2021). Local and non-local effects of foam rolling on passive soft tissue properties and spinal excitability. Front Physiol.

[CR38] Nakamura M, Onuma R, Kiyono R (2021). Acute and prolonged effects of different durations of foam rolling on range of motion, muscle stiffness, and muscle strength. Randomized Control Trial.

[CR39] Penichet-Tomas A, Pueo B, Abad-Lopez M, Jimenez-Olmedo JM (2021). Acute comparative effect of foam rolling and static stretching on range of motion in rowers. Sustain.

[CR40] Pepper TM, Brisme J-M, Sizer PS (2021). The Immediate Effects of Foam Rolling and Stretching on Iliotibial Band Stiffness: A Randomized Controlled Trial. Int J Sports Phys Ther.

[CR41] Power K, Behm D, Cahill F (2004). An acute bout of static stretching: effects on force and jumping performance. Med Sci Sports Exerc.

[CR42] Reid JC, Greene R, Young JD (2018). The effects of different durations of static stretching within a comprehensive warm-up on voluntary and evoked contractile properties. Eur J Appl Physiol.

[CR43] Reiner M, Tilp M, Guilhem G (2021). Effects of a single proprioceptive neuromuscular facilitation stretching exercise with and without post-stretching activation on the muscle function and mechanical properties of the plantar flexor muscles. Front Physiol to Be Printed.

[CR44] Reiner MM, Glashüttner C, Bernsteiner D (2021). A comparison of foam rolling and vibration foam rolling on the quadriceps muscle function and mechanical properties. Eur J Appl Physiol.

[CR45] Sagiroglu I, Kurt C, Pekünlü E, Özsu I (2017). Residual effects of static stretching and self-myofascial-release exercises on flexibility and lower body explosive strength in well-trained combat athletes. Isokinet Exerc Sci.

[CR46] Samson M, Button DC, Chaouachi A, Behm DG (2012). Effects of dynamic and static stretching within general and activity specific warm-up protocols. J Sports Sci Med.

[CR47] Škarabot J, Beardsley C, Štirn I (2015). Comparing the effects of self-myofascial release with static stretching on ankle range-of-motion in adolescent athletes. Int J Sports Phys Ther.

[CR48] Smith JC, Pridgeon B, Hall MC (2018). Acute effect of foam rolling and dynamic stretching on flexibility and jump height. J Strength Cond Res.

[CR49] Smith JC, Washell BR, Aini MF (2019). Effects of static stretching and foam rolling on ankle dorsiflexion range of motion. Med Sci Sports Exerc.

[CR50] Somers K, Aune D, Horten A (2020). Acute effects of gastrocnemius/soleus self-myofascial release versus dynamic stretching on closed-chain dorsiflexion. J Sport Rehabil.

[CR51] Su H, Chang NJ, Wu WL (2017). Acute effects of foam rolling, static stretching, and dynamic stretching during warm-ups on muscular flexibility and strength in young adults. J Sport Rehabil.

[CR52] Wilke J, Müller AL, Giesche F (2020). Acute effects of foam rolling on range of motion in healthy adults: a systematic review with multilevel meta-analysis. Sport Med.

[CR53] Wilson BR, Robertson KE, Burnham JM (2018). The relationship between hip strength and the y balance test. J Sport Rehabil.

[CR54] Yahata K, Konrad A, Sato S (2021). Effects of a high-volume static stretching programme on plantar-flexor muscle strength and architecture. Eur J Appl Physiol.

[CR55] Zaky HA, Mohamed MK, Barakat MH (2021). The effect of Foam Rolling and Dynamic Stretch on some physical abilities of female Handball Players. Int Sci J Phys Educ Sport Sci.

